# The Mystery of Dante Alighieri

**Published:** 1921-12

**Authors:** 


### THE REVIEW OF THE MONTH.

### THE MYSTERY OF DANTE ALIGHIERI.

DURING this year, the six hundredth since the death of the Florentine poet Dante Alighieri, much has been written about him, and all tends to accentuate the fact that he spoke after a fashion which has never yet been rightly apprehended.

The facts of his life have been very imperfectly recorded, but his writings form an intimate revelation of his personality which is of surpassing interest to the modern. He was born in Florence in 1265, became early celebrated as a writer of love songs, and published the mystic cycle of sonnets called "The New Life." He fought for his city, joined one of its Guilds, and began to take part in municipal affairs, though his chief interest lay in the study of philosophy. He married and had three children. In 1302 he was banished from Florence and was condemned to be burnt alive should he ever return. There is but scanty information of his doings after this. His last years were spent at Ravenna, and there he died on September 14, 1321.

From the point of view of worldly prosperity and success, the life of Dante was wrecked by the fierce struggle for supremacy between Guelfs and Ghibellines which inflamed the world for generations. The Ghibellines upheld the authority of the Holy Roman Empire, of which the ideal was a duly elected Emperor, functioning as head of a confederation of nations united under one Imperial law. The Guelfs claimed supreme lordship, no less in temporal than in spiritual affairs, for the Pope. In Dante's time both parties were gravely compromised, and for a long time he refused to belong officially to either. The Empire had degenerated into a predatory German principality; the Church had weakened its influence and dignity by an alliance with the unscrupulous Angevin invaders of Sicily and by perpetual interference with the civil rights of European sovereigns and the free cities of Italy.

Born of a Guelf family and deeply devoted to the Catholic faith, Dante gradually passed into a position of declared hostility to the Papacy. Successive Popes brought odium on the Curia. Boniface VIII., imbued with boundless lust for power, had compelled his predecessor, the saintly Celestine, to abdicate, and had subsequently, it was believed, compassed his death. A large secret party in the Church, including the poet, came to hold a conviction that the Holy See became vacant after Celestine died, and that all his successors were usurpers and enemies of the faith. The two French Popes, Clement V. and John XXII., did much to lend colour to this theory, and its consequences were remarkable. It dissolved allegiance to the Church, as then constituted, for the devout even more thoroughly than for the sceptic. The Popes being the foes of Christ, men were set free to criticise them and the ecclesiastics they put in power; to examine their doctrines, dispute their interpretation of Scripture, and repudiate their innovations.

Dante was among their boldest opponents. He wrote a very daring treatise in Latin denying the authority of the Decretals on which the autocratic power of the Popes was based, declaring in plain words that the vast possessions of the Church were ill-gotten and ought to be surrendered for the use of the poor, and that the claim of the Pope to universal dominion was indefensible and, indeed, absurd. He followed this up with "The Banquet," in which he attacked the popular theory that salvation is nowhere to be found save within the Church, and that all men who for any reason lack baptism are necessarily doomed to eternal perdition. He did this by painting the Divine radiance which rested on the Greek philosophers and the manifest acceptance at God's hand, attested by miracles, of the Roman people. In opposition to the ecclesiastical doctrine of man's inherent corruption and alienation from God, he demonstrated the nobility of man's nature with its Divine seed of Intellect imparted before birth and hence (by implication) before baptism.

Many notable examples of Dante's independence of thought might be quoted. We have only space for one. Nothing was more deeply rooted in the ecclesiastical system of the fourteenth century than contempt for the body. The monks and mystics of that age outdid each other in the injuries they inflicted on themselves with the notion of becoming thereby more acceptable to God. Loathsome dirt and vermin, hunger, thirst, cold, and torture were invited. God's gifts to the saint were sickness and pain. Health, beauty, and vigour were of the devil. Dante, in a beautiful chapter of "The Banquet," vindicating human nature, compares man's nobility to the skies in which God's gifts shine like stars, and goes on to enumerate the excellencies of the body imparted to it by Divine Love—"to wit, Beauty, Strength, and, so to speak, *perpetual Health.*" Had his sane and far-seeing philosophy prevailed, the world might have been spared six centuries of terrified fatalism and resistance to sanitation.

Later in life Dante wrote "The Divine Comedy," which purports to describe his journey through Hell, Purgatory, and Heaven. It is by the journey through Hell that the poet's grasp on the eternal verities is popularly judged. None have seemed to doubt that this crystallisation of a crude mediæval conception of God's mode with sinners was a reflection of Dante's inmost convictions. Yet its philosophy of life, death, and eternity is in striking contradiction to the testimony of his other works, and there is much in the "Purgatory," still more in the "Paradise," to awaken a doubt whether men have yet come to an understanding of the poet's purpose in lending this crowning work of his genius its mediæval setting. Surveyed in the light of his other works, the notion that Dante believed in such a hell as he depicted begins to lose ground.

